# Health-related quality of life of multiple sclerosis patients: a European multi-country study

**DOI:** 10.1186/s13690-021-00561-z

**Published:** 2021-03-20

**Authors:** Laurenske A. Visser, Celine Louapre, Carin A. Uyl-de Groot, William K. Redekop

**Affiliations:** 1 Department of Health Technology Assessment, Erasmus School of Health Policy & Management, Erasmus University Rotterdam, P.O.Box 1738/ 3000 DR, Rotterdam, The Netherlands; 2 ICM Institut du cerveau et de la moelle epiniere, Sorbonne University, APHP, F-75013 Paris, France; 3 Department of Neurology, Sorbonne University, APHP, Paris, France; 4 Bayle (J) Building, Burgemeester Oudlaan 50/ 3062, PA Rotterdam, The Netherlands

**Keywords:** Quality of life, Multiple sclerosis, Health utility, Disease-specific measure, EQ-5D

## Abstract

**Background:**

Inconsistent use of generic and disease-specific health-related quality of life (HRQOL) instruments in multiple sclerosis (MS) studies limits cross-country comparability. The objectives: 1) investigate real-world HRQOL of MS patients using both generic and disease-specific HRQOL instruments in the Netherlands, France, the United Kingdom, Spain, Germany and Italy; 2) compare HRQOL among these countries; 3) determine factors associated with HRQOL.

**Methods:**

A cross-sectional, observational online web-based survey amongst MS patients was conducted in June–October 2019. Patient demographics, clinical characteristics, and two HRQOL instruments: the generic EuroQOL (EQ-5D-5L) and disease-related Multiple Sclerosis Quality of Life (MSQOL)-54, an extension of the generic Short Form-36 (SF-36) was collected. Health utility scores were calculated using country-specific value sets. Mean differences in HRQOL were analysed and predictors of HRQOL were explored in regression analyses.

**Results:**

In total 182 patients were included (the Netherlands: *n* = 88; France: *n* = 58; the United Kingdom: *n* = 15; Spain: *n* = 10; living elsewhere: *n* = 11). Mean MSQOL-54 physical and mental composite scores (42.5, SD:17.2; 58.3, SD:21.5) were lower, whereas the SF-36 physical and mental composite scores (46.8, SD:22.6; 53.1, SD:22.5) were higher than reported in previous clinical trials. The mean EQ-5D utility was 0.65 (SD:0.26). Cross-country differences in HRQOL were found. A common predictor of HRQOL was disability status and primary progressive MS.

**Conclusions:**

The effects of MS on HRQOL in real-world patients may be underestimated. Combined use of generic and disease-specific HRQOL instruments enhance the understanding of the health needs of MS patients. Consequent use of the same instruments in clinical trials and observational studies improves cross-country comparability of HRQOL.

**Supplementary Information:**

The online version contains supplementary material available at 10.1186/s13690-021-00561-z.

## Background

Multiple sclerosis (MS) is a chronic disease with neurological dysfunction of the central nervous system, affecting 700,000 people in Europe and some 2.3 million people worldwide [1, 2]. While the survival of MS patients has improved [2], they still have lower health related quality of life (HRQOL) compared to the general population [3] and patients with other chronic diseases [4, 5]. HRQOL measures the impact of an illness or disease on the quality of life of patients as how they perceive it and its measurement helps to determine the effects of a treatment on HRQOL.

Many different generic or disease-specific HRQOL instruments have been used in MS-related randomized controlled trials (RCTs), observational studies and patient registries [6, 7]. Whilst generic instruments make it possible to compare HRQOL results to those of the general public and other diseases, disease-specific instruments can provide results that are more tailored to the disease in question [8]. Commonly used generic instruments in MS clinical studies and economic evaluations are the Medical Outcomes Study Short Form-36 (SF-36) and the EuroQOL 5 dimensions questionnaire (EQ-5D) [6, 8–10]. A hybrid HRQOL instrument that combines aspects of the generic SF-36 with MS-specific domains, such as questions about bladder/bowel function and sexual function, is the Multiple Sclerosis Quality of Life (MSQOL)-54 instrument [11]. While it is advisable to use both generic and disease-specific instruments to inform health technology assessment of treatments [12], it is uncommon to include both instruments in RCTs or observational studies [6]. To be able to inform both healthcare professionals and policy makers about the HRQOL of MS patients outside a clinical setting, it is therefore interesting to collect so-called real-world data.

The use of diverse HRQOL instruments in different MS studies limits the comparability of outcomes between these studies [13]. To the best of our knowledge, there has been no recent comprehensive European study of the HRQOL of MS patients in a real-world setting using both a generic and disease-specific HRQOL instrument. The aim of this study is threefold: 1.) to investigate the real-world HRQOL of patients with MS in several European countries including the Netherlands, France, the United Kingdom, Spain, Germany and Italy, 2.) to compare HRQOL among these countries, and 3.) determine factors associated with HRQOL.

## Methods

### Study design

We performed a cross-sectional online survey between June and October 2019 in six European countries (the Netherlands, France, the United Kingdom, Spain, Germany and Italy). These countries were selected to give a representative overview of the HRQOL of the EU-5 and the Dutch MS patient population. Patients were recruited through the information channels of national patient societies and social media. Based on feasibility and the exploratory nature of the study (to investigate the current HRQOL of MS patients, and no hypothesis testing) we aimed to include 50 persons per country (i.e. non-probability sampling). In France the patients were also recruited by a MS specialist working at a MS centre in Paris. The study information and informed consent form could be downloaded by the patient. The research protocol was reviewed and approved by the Medical Research Ethics Committees of the Erasmus Medical Centre (MEC-2018-1636). Qualtrics XM software was used to perform the survey. The STROBE checklist for cross-sectional studies was used for reporting this study [14].

### Inclusion criteria

Patients had to be ≥18 years old and have the diagnosis of clinically definite MS (including clinically isolated syndrome (CIS), relapsing-remitting MS (RRMS), secondary progressive MS (SPMS), or primary progressive MS (PPMS)). No restrictions were made on whether patients were currently taking or had previously taken disease modifying therapies (DMTs). Participants required access to the internet.

### Data collection

The online web-based survey consisted of three parts: 1) patient demographics and clinical characteristics; 2) the EQ-5D with five levels (EQ-5D-5L) to collect information about the patient’s health state [10]; and 3) the MSQOL-54 to collect information about the disease-related QOL [11]. The survey took roughly 15–20 min to complete.

The survey was available in six languages and the participant was free to choose which language to use when completing it. Questions regarding demographics and clinical characteristics were translated from Dutch into the other languages by a professional translation agency and double-checked by native speakers. Official translations of the EQ-5D-5L and the MSQOL-54 were used.

### Measures

Patients were asked to provide information on their age, country of residence (options: the Netherlands, France, the United Kingdom, Spain, Germany, Italy and ‘living elsewhere’), nationality, gender, marital status and educational level. Clinical characteristics included the type of MS, age at diagnosis, disability and their current and previous treatment. Disability was self-reported using the Expanded Disability Status Scale (EDSS), an instrument to rate neurological impairments, with a total score ranging from 0 (normal) to 10 (death due to MS) [15].

The EQ-5D-5L is a standardized and validated HRQOL instrument [10, 16] that yields a single generic measure of health to quantify HRQOL used in clinical and economic evaluations [6, 17]. The health states can be converted into a single “health utility” score, where 0 equals death and 1.0 equals perfect health. For France, the Netherlands and Spain the health utility scores were calculated using French, Dutch and Spanish country value sets, respectively [18–20]. For the UK, the crosswalk value set was used [21, 22]. The value set used to calculate utility scores of patients living elsewhere was determined by the most commonly used language filled in by the patients living elsewhere.

The MSQOL-54 is a validated instrument with an adequate test-retest reliability, construct validity and internal consistency [11]. The instrument consists of the generic SF-36 [9], extended with health concepts relevant for MS patients [11]. It contains 52 QOL items that are divided across 12 scales (physical function, role limitations-physical, role limitations-emotional, pain, emotional well-being, energy, health perceptions, social function, cognitive function, health distress, overall quality of life, and sexual function) and two single items (satisfaction with sexual function and change in health) [11]. Two summary scores, physical health composite score (PHCS) and mental health composite score (MHCS) are derived from a weighted combination of scale scores. Scale and composite scores range from 0 to 100, where a higher score indicates a better QOL [11]. The SF-36 composite scores can be calculated from the MSQOL-54 [11] and have a mean of 50 (SD:10) in the general population [23].

### Statistical analysis

Statistical analyses were performed with Stata 16.0. Analyses were stratified according to country of residence. Differences in mean scores across countries were calculated using the analysis of variance (ANOVA) test; if assumptions for the ANOVA were violated (we checked the distribution of the variables by plotting a histogram), the non-parametric Kruskal-Wallis test was used. Testing for a relationship between categorical variables was done using the chi-square test or the non-parametric Fishers exact test for small samples. Post-hoc analysis was done using either the Bonferroni correction or the Dunn’s test. Statistical significance was defined as *p* < 0.05.

Bivariate association of the EQ-5D-5L dimensions to EDSS was investigated with Spearman correlation coefficients. Univariate and multivariate analyses were conducted to examine the relationship between patient demographics and the EQ-5D-5L dimensions, health utility, the MSQOL-54 scales and the PHCS/MHCS. Covariates included in the multiple regressions were based on significance in the univariate analysis. A linear or logistic regression was performed for a continuous or categorical outcome variable, respectively. The stepwise backward selection method was used for the multivariate regression and variables were kept in the model based on significance.

## Results

The attempted sample size of 50 per country was not feasible in the UK, Spain, Germany and Italy given that patients were very difficult to recruit, therefore we ceased recruitment 5 months after the start of the study. A total of 281 patients were recruited and started the survey. Ninety-nine participants were dropped from the analysis since they did not finish the survey (*n* = 55), did not give informed consent (*n* = 39), had provided an age of diagnosis that was older than their current age (*n* = 3), or were younger than 18 years (*n* = 2). This left 182 participants for analysis.

Patient demographics can be found in Table 1. Patients were analysed based on country of residence (the Netherlands: *n* = 88; France: *n* = 58; the United Kingdom: *n* = 15; living elsewhere: *n* = 11; Spain: *n* = 10). The total population had a median age of 43 years old and the average age at diagnosis was 34 years old. Roughly 80% were female and most participants (80%) had RRMS. There were no significant differences in patient demographics across the countries other than the age at diagnosis (range: 31.2–41.6 years) and educational level (an average of 52% having a university degree).

**Table 1:** Patient demographics (mean, standard deviation, number, frequency and *p*-value) of the total study population (*n* = 182) and per country (the Netherlands, France, the United Kingdom, Spain and elsewhere (Germany or Italy)), 2019

	Total (***n*** = 182)	The Netherlands (***n*** = 88)	France (***n*** = 58)	The United Kingdom (***n*** = 15)	Spain (***n*** = 10)	Elsewhere (***n*** = 11)	***P***-value
**Age, mean (±SD)**	43.09 (10.53)	43.97 (10.27)	40.88 (10.57)	47.6 (9.39)	42.90 (11.37)	41.73 (12.08)	0.237^a^
**Age at diagnosis, mean (±SD)**	34.12 (10.36)	35.18 (10.3)	31.22 (9.53)	41.6 (10.17)	31.67 (11.04)	32.75 (9.84)	0.006^a^*
**Time since diagnosis, mean (±SD)**^**c**^	8.97 (7.8)	8.78 (7.33)	9.66 (8.61)	6 (4.31)	9.8 (9.5)	10.18 (927)	0.834^a^
**Gender, n (%)**							0.746^b^
Male	39 (21.43)	21 (23.86)	9 (15.52)	3 (20.00)	3 (33.00)	3 (27.27)	
Female	142 (78.02)	66 (75.00)	49 (84.48)	12 (80.00)	7 (70.00)	8 (72.73)	
Prefer not to say	1 (0.55)	1 (1.14)					
**Type of MS, n (%)**							0.649^b^
CIS	2 (1.10)		2 (3.45)				
RRMS	146 (80.22)	70 (79.55)	47 (81.03)	10 (66.67)	9 (90.00)	10 (90.91)	
PPMS	17 (9.34)	10 (11.36)	4 (6.90)	3 (20.00)	1 (10.00)	1 (9.09)	
SPMS	17 (9.34)	8 (9.09)	5 (8.62)	2 (13.33)			
**EDSS, n (%)**							0.070^b^
EDSS <= 2.5	45 (24.73)	12 (13.64)	20 (34.48)	3 (20.00)	6 (60.00)	4 (36.36)	
EDSS 3–6.5	35 (19.23)	15 (17.05)	12 (20.69)	4 (26.67)	2 (20.00)	2 (18.18)	
EDSS > = 6	26 (14.29)	14 (15.91)	6 (10.34)	6 (40.00)	2 (20.00)		
Unknown	58 (31.87)	30 (34.09)	20 (34.48)	2 (13.33)		4 (36.36)	
Missing	18 (9.89)	17 (19.32)				1 (9.09)	
**Marital status, n (%)**							0.986^b^
Single	36 (19.78)	17 (19.32)	13 (21.41)	3 (20.00)	1 (10.00)	2 (18.18)	
Partnered	42 (23.08)	21 (23.86)	13 (21.41)	5 (33.33)	2 (20.00)	1 (9.09)	
Married	96 (52.75)	44 (50.00)	30 (51.72)	7 (46.67)	7 (70.00)	8 (72.73)	
Divorced	7 (3.85)	5 (5.68)	2 (3.45)				
Widowed	1 (0.55)	1 (1.14)					
**Educational level, n (%)**							0.000^b^
Primary education	1 (0.55)				1 (10.00)		
Secondary education	13 (7.14)	9 (10.23)		3 (20.00)		1 (9.09)	
Vocational/technical education	67 (36.82)	44 (50.00)	14 (24.14)	4 (26.67)	3 (30.00)	2 (18.18)	
University	95 (52.20)	35 (39.77)	41 (70.69)	6 (40.00)	5 (50.00)	8 (72.73)	
Other	6 (3.30)		3 (5.17)	2 (13.33)	1 (10.00)		
**Nationality, n (%)**							
British	14 (7.69)			14 (93.33)			
French	59 (32.42)		57 (98.28)			2 (18.18)	
Dutch	91 (50.00)	87 (98.86)	1 (1.72)			3 (27.27)	
Spanish	9 (4.95)				9 (90.00)		
Other	9 (4.95)	1 (1.14)		1 (6.67)	1 (10.00)	6 (54.55)	

*MS* Multiple sclerosis, *CIS* Clinically isolated syndrome, *RRMS* Relapsing-remitting MS, *PPMS* Primary progressive MS, *SPMS* Secondary progressive MS, *EDSS* Expanded Disability Status Scale, ^a^: Kruskal-Wallis test, ^b^: Fisher’s exact test, ^c^: Time since diagnosis was calculated by subtracting the age at diagnosis from the current age, *Significant difference in mean age at diagnosis between France and the United Kingdom (*p* = 0.002) using Dunn’s test and Bonferroni correction

The results regarding current and past treatments can be found in the Additional file 1 (Table A1). A total of 165 patients (90.8%) received DMT at one time or another; more than two-thirds were either currently taking a DMT (*n* = 131, 72.0%) or had received it in the past (*n* = 34, 18.9%). Of those currently taking a DMT, 44.7% were taking a first-line DMT, which was either an injectable (*n* = 26; 19.9%) or an oral treatment (*n* = 32; 24.4%). Over half of the patients currently taking a DMT were receiving a second-line therapy (*n* = 73; 55.7%). A minority received oral treatment (*n* = 20; 27.4%) and the majority received infusion therapy (*n* = 52; 71.2%). Many of the patients receiving infusion therapy were taking ocrelizumab (*n* = 39; 75%).

The DMT frequencies seen in the different countries were not significantly different, except for the use of INF-β 1a (*p* = 0.018) and ocrelizumab (*p* = 0.003) (Table A1). A cross-country difference was found regarding previous use of INF-β 1a (range: 6.7–27.6%), driven primarily by the French and Dutch populations. Furthermore, a cross-country difference was found regarding current use of ocrelizumab (range: 0.0–32.9%). Treatments used by less than 5% of the patients included INF-β 1b, cladribine and alemtuzumab.

### EQ-5D-5L

The total mean health utility score was 0.65 (SD:0.26) (Table 2). Overall, one-third of all patients had moderate problems with mobility (*n* = 57; 31.3%) and 44% had moderate problems with usual activities. A majority had slight to moderate pain and discomfort (*n* = 62; 34.17% and *n* = 60; 32.9%). Generally, the patients had no problems with self-care (*n* = 117; 64.3%) or anxiety and depression (*n* = 75; 41.2%).

**Table 2:** Problems in the EQ-5D-5L dimensions and health utility (mean, standard deviation, number, frequency and p-value) of the total study population and according to country of residence (the Netherlands, France, the United Kingdom, Spain and elsewhere (Germany or Italy)), 2019

	Total (***n*** = 182)	The Netherlands (***n*** = 88)	France (***n*** = 58)	The United Kingdom (***n*** = 15)	Spain (***n*** = 10)	Elsewhere (***n*** = 11) ^**c**^	***P***-value
**Mobility, n (%)**							0.086^b^
No problems	47 (25.82)	15 (17.05)	21 (36.21)	1 (6.67)	6 (60.00)	4 (36.36)	
Slight problems	45 (24.73)	25 (28.41)	12 (20.69)	3 (20.00)	1 (10.00)	4 (36.36)	
Moderate problems	57 (31.32)	29 (32.95)	16 (27.59)	6 (40.00)	3 (30.00)	3 (27.27)	
Severe problems	29 (15.93)	17 (19.32)	7 (12.07)	5 (33.33)			
Unable to walk	4 (2.20)	2 (2.27)	2 (3.45)				
**Self-care, n (%)**							0.094^b^
No problems	117 (64.29)	50 (56.82)	44 (75.86)	5 (33.33)	9 (90.00)	9 (81.82)	I
Slight problems	38 (20.88)	21 (23.86)	10 (17.24)	4 (26.67)	1 (10.00)	2 (18.18)	
Moderate problems	20 (10.99)	12 (13.64)	3 (5.17)	5 (33.33)			
Severe problems	4 (2.20)	3 (3.41)		1 (6.67)			
Unable to wash or dress myself	3 (1.65)	2 (2.27)	1 (1.72)				
**Usual activities, n (%)**							0.254^b^
No problems	28 (15.38)	10 (11.36)	11 (18.97)	1 (6.67)	3 (30.00)	3 (27.27)	
Slight problems	44 (24.18)	19 (21.59)	16 (27.59)	2 (13.33)	4 (40.00)	3 (27.27)	
Moderate problems	80 (43.96)	39 (44.32)	27 (46.55)	8 (53.33)	2 (20.00)	4 (36.36)	
Severe problems	27 (14.84)	18 (20.45)	4 (6.90)	3 (20.00)	1 (10.00)	1 (9.09)	
Unable to do my usual activities	3 (1.65)	2 (2.27)		1 (6.67)			
**Pain / discomfort, n (%)**							0.529^b^
No pain	27 (14.84)	17 (19.32)	5 (8.62)	1 (6.67)	3 (30.00)	1 (9.09)	
Slight pain	62 (34.07)	27 (30.68)	23 (39.66)	4 (26.67)	3 (30.00)	5 (45.45)	
Moderate pain	60 (32.97)	25 (28.41)	21 (36.21)	6 (40.00)	4 (40.00)	4 (36.36)	
Severe pain	29 (15.93)	18 (20.45)	7 (12.07)	3 (20.00)		1 (9.09)	
Extreme pain	4 (2.20)	1 (1.14)	2 (3.45)	1 (6.67)			
**Anxiety / depression, n (%)**				.			0.061^b^
No problems	75 (41.21)	48 (54.55)	14 (24.14)	6 (40.00)	4 (40.00)	3 (27.27)	
Slight problems	50 (27.47)	19 (21.59)	21 (36.21)	4 (26.67)	3 (30.00)	3 (27.27)	
Moderate problems	37 (20.33)	14 (15.91)	12 (20.69)	4 (26.67)	2 (20.00)	5 (45.45)	
Severe problems	17 (9.34)	7 (7.95)	8 (13.79)	1 (6.67)	1 (10.00)		
Extremely anxious/depressed	3 (1.65)		3 (5.17)				
**Health utility, mean (SD)**	0.65 (0.26)	.58 (0.28)	.76 (0.22)	0.48 (0.25)	0.78 (0.14)	0.68 (0.19)	< 0.001^a^*

^a^ Kruskal-Wallis test, ^b^ Fisher’s exact test, *SD* Standard deviation ^c^ Index calculated with the Dutch tariff since *n* = 8/11 filled out the Dutch version of the survey. *: Significant difference in health utility scores between France and the Netherlands (*p* < 0.001), France and the United Kingdom (*p* < 0.001), the United Kingdom and Spain (*p* = 0.021), based on Dunn’s test and Bonferroni correction

Given the country-specific tariffs, there were statistically significant differences in utility between the countries (range: 0.48–0.78; *p* < 0.001) (Table 2). However, once calculated using only the Dutch tariff, this was no longer the case (range: 0.48–0.73; *p* = 0.012) (results not shown). No statistical between-country differences were found amongst the EQ-5D-5L dimensions (Table 2).

**Fig. 1:**
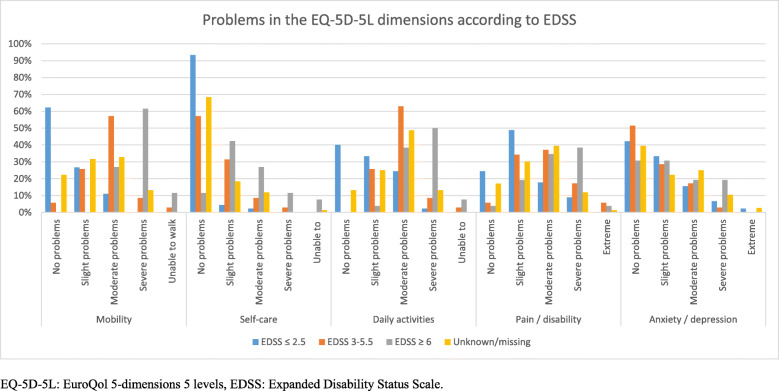
Problems in the EQ-5D-5L dimensions according to disability status of the total study population (the Netherlands, France, the United Kingdom, Spain and elsewhere (Germany or Italy)), 2019. EQ-5D-5L: EuroQol 5-dimensions 5 levels, EDSS: Expanded Disability Status Scale

Patients with mild disability (EDSS ≤2.5) generally had no problems with mobility (*n* = 28; 62.2%) or self-care (*n* = 42; 93.3%) (Fig. 1). However, almost 60% had slight to moderate problems with daily activities, and 48.9% suffered from slight pain. The majority were not anxious or depressed (*n* = 19; 42.2%). Patients with greater disability (EDSS 3–5.5 and ≥ 6) were more likely to have moderate to severe problems in mobility, daily activities and pain/discomfort. However, disability was not associated with anxiety/depression. Furthermore, there was a strong and significant correlation of the EQ-5D-5L domains mobility, self-care and usual activities to disability (Additional file 1: Table A2). Pain/ discomfort had a moderate significant correlation, whereas anxiety/depression had a weak though non-significant correlation to disability.

### MSQOL-54 and SF-36

The results of the MSQOL-54 and SF-36 are presented in Table 3. The mean MSQOL-54 physical health composite score (MSQOL-54 PHCS) and mental health composite scale (MSQOL-54 MHCS) for the total population was 42.5 (SD: 17.2) and 58.3 (SD: 21.5), respectively. The mean MSQOL-54 PHCS differed significantly between countries (range: 31.9–55.6, *p* = 0.017). In contrast, no significant difference between countries was found on the MSQOL-54 MHCS (range: 51.9–65.9, *p* = 0.06). The mean SF-36 physical composite score (SF-36 PCS) and mental composite score (SF-36 MCS) for the total population was 46.8 (SD: 22.6) and 53.1 (SD: 22.45), respectively. Both scores differed significantly between countries (PCS: range 32.9–65.1, *p* = 0.007; MCS: range 44.5–65.7, *p* = 0.016).

**Table 3:** The MSQOL-54 and SF-36 scores (mean, standard deviation and p-value) of the total study population and according to country of residence (the Netherlands, France, the United Kingdom, Spain and elsewhere (Germany or Italy)), 2019

	Total (***n*** = 182)	The Netherlands (***n*** = 88)	France (***n*** = 58)	The United Kingdom (***n*** = 15)	Spain (***n*** = 10)	Elsewhere (***n*** = 11)	***P***-value ^**a**^
***MSQOL-54***							
**Physical health composite score, mean (SD)**	**42.54 (17.15)**	**42.07 (16.72)**	**42.14 (16.73)**	**31.89 (15.18)**	**55.65 (16.98)**	**49.97 (17.61)**	0.017^I^
Physical function	54.86 (30.63)	49.55 (29.43)	63.85 (30.35)	28.74 (26.65)	72.00 (18.74)	70.00 (24.49)	< 0.000^II^
Health perception	38.8 (22.56)	38.42 (24.19)	35.93 (18.73)	34.53 (18.59)	54.17 (22.62)	48.75 (27.48)	0.177
Energy/ fatigue	35.88 (19.86)	38.95 (20.79)	30.41 (17.51)	27.47 (12.18)	44.20 (21.05)	44.00 (22.77)	0.022
Role limitations – physical	30.77 (37.35)	26.04 (34.06)	35.06 (39.70)	16.67 (30.86)	60.00 (44.41)	38.64 (39.31)	0.043^III^
Pain	65.81 (25.7)	68.22 (25.48)	63.16 (26.08)	53.89 (30.1)	72.00 (15.03)	71.06 (24.18)	0.388
Sexual function combined	65.89 (27.13)	70.17 (26.65)	60.49 (24.76)	49.4 (32.44)	76.67 (26.88)	71.22 (26.45)	0.013
Sexual function male	65.57 (29.27)	63.34 (30.64)	68.53 (26.61)	47.23 (41.12)	80.56 (26.79)	75.01 (25.00)	0.769
Sexual function female	65.97 (26.64)	72.19 (25.26)	59.01 (24.41)	50 (32.06)	75.00 (28.87)	69.80 (28.50)	0.008^IV^
Health distress	54.95 (24.59)	56.33 (24.56)	50.34 (24.88)	53 (22.82)	69.50 (28.03)	57.73 (19.02)	0.251
**Mental health composite score, mean (SD)**	**58.26 (21.48)**	**62.15 (19.89)**	**51.93 (22.34)**	**54.98 (23.1)**	**65.98 (25.00)**	**57.64 (17.83)**	0.060^V^
Health distress	54.95 (24.59)	56.33 (24.56)	50.34 (24.88)	53 (22.82)	69.50 (28.03)	57.73 (19.02)	0.251
Overall quality of life	58.51 (19.06)	60.98 (18.48)	55.83 (20.07)	48.11 (19.05)	64.08 (14.94)	61.29 (17.08)	0.082
Emotional well-being	62.53 (21.01)	68.09 (18.45)	53.36 (22.06)	60 (22.32)	66.00 (24.96)	66.64 (13.84)	0.003^VI^
Role limitations – emotional	57.04 (43.51)	63.22 (42.54)	47.95 (42.73)	53.33 (46.80)	70.00 (42.89)	48.48 (47.99)	0.195
Cognitive function	54.52 (24.85)	56.19 (24.06)	51.18 (23.85)	58.00 (32.34)	58.50 (23.46)	50.45 (28.24)	0.610
**Change in health**	44.23 (26.29)	47.16 (29.47)	43.97 (23.09)	31.67 (19.97)	52.5 (27.51)	31.82 (11.68)	0.079
**Satisfaction with sexual function**	52.63 (33.47)	51.42 (36.03)	51.29 (28.65)	40.38 (36.14)	72.5 (34.26)	65.91 (25.67)	0.121
***SF-36***							
**Physical composite scale, mean (SD)**	46.82 (22.60)	44.71 (21.25)	48.56 (22.30)	32.90 (19.38)	65.10 (22.34)	56.9 (26.05)	0.007^VII^
**Mental composite scale, mean (SD)**	53.11 (22.45)	57.02 (21.09)	46.40 (22.57)	44.54 (22.23)	65.69 (25.22)	57.25 (20.17)	0.016^VIII^

*MSQOL-54* Multiple Sclerosis Quality of Life −54 instrument, *SF-36* Medical Outcomes Study Short Form-36. ^a^ Kruskal-Wallis test and post-hoc analysis using Dunn’s test and Bonferroni correction. ^I^ Significant difference in mean physical health composite score between the United Kingdom and Spain (*p* = 0.007); ^II^ Significant difference in mean physical function scores between the United Kingdom and France, the United Kingdom and elsewhere and the United Kingdom and Spain (*p* = < 0.000 for all three situations), and France and the Netherlands (*p* = 0.04); ^III^ Significant differences in mean role limitations physical between the United Kingdom and Spain (*p* = 0.029); ^IV^ Significant difference in mean sexual function female between France and elsewhere (*p* = 0.014); ^V^ Significant difference in mean mental health composite score between France and the Netherlands (*p* = 0.046); ^VI^ Significant difference in mean emotional well-being score between France and the Netherlands (*p* < 0.000); ^VII^ Sig difference between the United Kingdom and Spain (*p* = 0.003), almost reaching significance difference between the United Kingdom and elsewhere (*p* = 0.057), and the Netherlands and Spain (*p* = 0.059); ^VIII^ Almost reaching sig difference between France and Spain (*p* = 0.058). The sample size varied somewhat across the scales and the scores were calculated by excluding missing data [11]. For 27 questions the range of missing data was 0.55–2.75% (*n* = 1 to *n* = 5). Additionally, the range of missing data for four out of five health perception scale questions was higher (*n* = 20 to *n* = 54; 14.83–29.67%)

Missing data: The sample size varied somewhat across the domains, the composite scores were calculated from the domains by excluding missing data. Missing data were found in physical health function question 3 (0.55%, *n* = 1 from the UK), physical health function question 5 (0.55%, *n* = 1 from FR), physical health function question 8 (1.65%, *n* = 3 from the NL), physical health function question 10 (0.55%, *n* = 1 from the NL), health perceptions question 34 (25.27%, *n* = 4, *n* = 7, *n* = 31, *n* = 2, and *n* = 2 from the UK, FR, NL, SP and elsewhere, respectively), health perceptions question 35 (10.99%, n = 3, *n* = 6, *n* = 9, *n* = 2 from the UK, FR, NL and SP country of residence, respectively), health perceptions question 36 (29.67%, n = 3, *n* = 15, *n* = 30, *n* = 4, *n* = 2 from the UK, FR, NL, SP and elsewhere, respectively), health perceptions question 37 (14.83%, *n* = 2, *n* = 12, *n* = 11, *n* = 4, *n* = 2 from the UK, FR, NL and SP country of residence, respectively), energy/fatigue question 23 (0.55%, *n* = 1 from SP), energy/fatigue question 27 (0.55%, *n* = 1 from FR), energy/fatigue question 29 (1.10%, *n* = 1, *n* = 1 from FR and NL respectively), energy/fatigue question 32 (1.65%, *n* = 3 from FR), role limitations physical question 13 (1.10%, *n* = 2 from FR), role limitations physical question 14 (0.55%, *n* = 2 from NL), role limitations physical question 15 (0.55%, *n* = 1 from NL), role limitations physical question 16 (1.10%, *n* = 2 from NL), sexual function male (0.55%, *n* = 1 from NL), sexual function female (0.55%, *n* = 1 from UK), satisfaction with sexual function (1.10%, *n* = 2 from UK), health distress question 39 and 40 (0.55%, *n* = 1 from NL in both cases), overall quality of life (0.55%, *n* = 1 from FR), emotional well-being question 28 (2.20%, *n* = 1, *n* = 2, *n* = 1 from FR, NL and elsewhere, respectively), emotional well-being question 30 (0.55%, *n* = 1 from FR), role limitations emotional question 17 (1.10%, *n* = 1, *n* = 1 from FR and NL, respectively), role limitations emotional question 18 (1.65%, *n* = 2, *n* = 1 from FR and NL, respectively), role limitations emotional question 19 (2.20%, *n* = 2, *n* = 2 from FR and NL, respectively), cognitive function question 42 (0.55%, *n* = 1 from SP), cognitive function question 43 (1.10%, *n* = 2 from ES), cognitive function question 44 (1.65%, *n* = 1, *n* = 1, *n* = 1 from FR, NL and SP, respectively), cognitive function question 45 (2.75%, *n* = 1, *n* = 4 from FR and SP, respectively).

### Regression analyses

Table 4 shows the results of the analyses to examine the relationship between patient demographics and the health utility, PHCS and MHCS scores. Multivariate analysis found that age, age at diagnosis, marital status and current line of treatment were not associated with utility, PHCS and MHCS. PPMS was independently associated with lower utility, PHCS and MHCS. Furthermore, moderate to severe disability (EDSS 3–9.5) and unknown disability was independently associated with lower utility and PHCS. After correction for other characteristics, French patients reported a higher utility than other patients, while Dutch and Spanish patients reported a higher PHCS score.

**Table 4:** Linear regression estimates: predictors of health utility and the MSQOL-54 composite scores of the total study population (the Netherlands, France, the United Kingdom, Spain and elsewhere (Germany or Italy)), 2019

	Health utility Univariate	Health utility Multivariate ^a^	PHCS Univariate	PHCS Multivariate	MHCS Univariate	MHCS Multivariate
**Age (years)**	−0.006 (0.002)***		−0.257 (0.120)**		0.149 (0.154)	
**Age at diagnosis (years)**	−0.007 (0.002)**		−0.159 (0.124)		−0.010 (0.156)	
**Time since diagnosis (years)**	−0.000 (0.003)		−0.190 (0.163)		0.282 (0.205)	
**Gender (female vs. male)**	0.055 (0.047)		0.482 (3.098)		−3.742 (3.883)	
**Marital status**						
Single						
Partnered	0.119 (0.061) *		3.770 (3.923)		6.816 (4.958)	
Married	0.084 (0.052)		5.372 (3.384)		7.889 (4.289)*	
Divorced	−0.070 (0.110)		−4.081 (7.097)		9.444 (8.921)	
Widowed	0.171 (0.280)		−3.549 (17.383)		16.736 (21.807)	
**Educational level**						
Primary education						
Secondary education	−0.296 (0.274)		−38.651 (17.450)**		−23.068 (22.460)	
Vocational/technical education	−0.223 (0.266)		−33.782 (16.940)**		−25.104 (21.803)	
University	−0.119 (0.265)		−27.890 (16.905)		−24.458 (21.759)	
Other	−0.160 (0.285)		−32.622 (18.163)*		−24.722 (23.377)	
**Country of residence**						
United Kingdom						
France	0.281 (0.073)***	0.131 (0.034)***	10.251 (4.968)**		−3.051 (6.137)	
The Netherlands	0.098 (0.070)		10.174 (4.800)**	5.364 (2.445)**	7.168 (5.912)	
Spain	0.293 (0.103)***		23.758 (6.907)***	11.074 (5.002)***	11.000 (8.633)	
Elsewhere	0.195 (0.100)*		18.075 (6.722)***		2.663 (8.395)	
**Type of MSI**						
CIS / RRMS						
PPMS	−0.387 (0.062)***	−0.215 (0.058)***	−21.055 (4.219)***	−13.024 (4.208)***	−17.066 (5.343)***	−17.815 (5.326)***
SPMS	−0.143 (0.062)**		−9.204 (4.105)**		7.183 (5.343)	
**EDSS**						
EDSS <= 2.5						
EDSS 3–5.5	−0.161 (0.051)***	−0.129 (0.048)***	−12.436 (3.482)***	−11.320 (3.419)***	− 1.415 (4.847)	
EDSS > = 6	−0.485 (0.056)***	− 0.383 (0.056)***	−25.362 (3.854)***	−21.194 (3.997)***	−7.789 (5.294)	
Unknown / missing	−0.151 (0.043)***	−0.113 (0.040)***	−11.534 (2.906)***	−10.677 (2.851)***	−7.121 (4.064)*	
**Current line of treatment**						
Treatment naive						
1st-line DMT	0.259 (0.071)***		10.778 (4.785)**		1.709 (5.970)	
2nd-line DMT	0.115 (0.069)		4.477 (4.677)		1.259 (5.844)	
Treatment experience but currently no DMT	0.127 (0.077)*		4.857 (5.137)		3.450 (6.429)	

*PHCS* Physical health composite score, *MHCS* Mental health composite score, *CIS* Clinically isolated syndrome, *RRMS* Relapsing-remitting multiple sclerosis, *PPMS* Primary progressive multiple sclerosis *SPMS* Secondary progressive multiple sclerosis, *EDSS* Expanded Disability Status Scale, *DMT* Disease modifying therapy. ^a^ Results show the regression with utility calculated using the country-specific tariffs. The multivariate regression was re-run calculating the utility with only the Dutch tariffs; country of residence was no longer a significant variable (Additional file 1 Table A3)

*** p < 0.01, ** p < 0.05, * p < 0.1 Standard errors are in parenthesis

Additional univariate and multivariate models for the EQ-5D-5L dimensions and utility, MSQOL-54 scales and composite scores are shown in the Additional file 1 (Tables A3, A4).

## Discussion

The aim of this study was to examine real-world HRQOL of patients with MS in several European countries. The generic health utility instrument (the EQ-5D-5L) and the hybrid disease-specific MSQOL-54 (including the SF-36) instruments were used to calculate HRQOL. Compared to previous research, our results indicate that the HRQOL of MS patients may have been overestimated. We found a relatively low health utility score, with no between-country differences amongst the EQ-5D-5L dimensions. Somewhat low HRQOL was found using the MSQOL-54 with between-country differences. Furthermore, disability status and PPMS is negatively correlated with HRQOL.

The mean EQ-5D utility score (0.65 ± 0.26 SD) in our population was lower than the scores (range: 0.69–0.78) reported in other multi-country studies [24–26]. Previous MS studies have used the older EQ-5D-3L method, rather than the newer EQ-5D-5L [24–27]. However, the EQ-5D-5L significantly increases sensitivity, reliability and has less of a ceiling effect than the EQ-5D-3L [16]. Given that previous studies used the older EQ-5D-3L method to calculate utilities this limits the comparability. Nonetheless, a similar trend is seen regarding disability and problems experienced in the dimensions. For example, the correlation of EQ-5D-3L domains to EDSS found by the European observational study by Eriksson et al. (2019) are of the same magnitude to ours (the coefficients for mobility: 0.77 vs 0.83; self-care 0.67 vs 0.68; usual activities: 0.64 vs 0.73; pain/discomfort: 0.37 vs 0.46; anxiety/depression: 0.13 vs 0.06) [27]. As such, patients are more likely to suffer from problems with mobility, self-care, daily activities and pain/discomfort with increasing disability. Regarding anxiety and depression, MS patients did not seem to experience increasing problems with increasing disability.

Our European population had a somewhat higher mean SF-36 physical component summary score (46.8 SE:1.7) and mental component summary score (53.11 SE:1.6) than most previously published DMT studies. The CONFIRM and DEFINE study, examining HRQOL while taking dimethyl fumarate, found mean SF-36 PCS and MCS of 43.1 and 47.2, and 43.4 and 45.3, respectively [24, 25]. Also, the CARE-MS I and II trials (treatment naïve and treatment experienced patients receiving either INF-β 1a or alemtuzumab), found SF-36 composite scores higher than our study (PCS range: 43.9–46.5; MCS range: 42.4–48.3) [26]. Even after the use of DMT treatment, the trials found lower composite scores compared to our results [24–26]. This is somewhat in contrast to the review article by Jongen (2017), whom found that in clinical trials and observational studies DMT treatment may have a positive effect on HRQOL in RRMS patients [6], however it is less clear what the HRQOL is after such interventions. This may suggest that, based on the SF-36, real-world HRQOL of patients is more favourable than during a clinical trial.

The mean MSQOL-54 composite scores (PHCS: 42.5 SE:1.3; MHCS: 58.2 SE:1.6) in our European population were 10–20 points lower than scores reported in previous observational studies [28–32]. For example, a European observational phase 4 study including 284 patients from the Netherlands, Belgium, Luxembourg and the United Kingdom, found mean MSQOL-54 PHCS and MHCS scores of 56.6 and 57.2 [32]. Lower scores to ours may be due to population differences. The phase 4 study had a younger patient population (mean age 38.6 vs 43.1 years), less time since their RRMS diagnosis (3.5 vs 8.9 years) and less disability (mean EDSS 2.4 vs 3.5). Moreover, a 10-year HRQOL observational study with 77 ambulatory MS patients in Finland found PHCS of 63.9 (SE:2.1) and MHCS 73.6 (SE: 2.2) [31]. Again, differences may be due to patient demographics, while the patients included in Finland were somewhat older at baseline (mean age 47 vs 43 years old), the majority of patients had less disability (70% of patients had EDSS 0–3).

Disease-specific measurements are more extensive and have greater sensitivity to changes, meaning it can detect HRQOL differences between patients [6]. When correcting for patient characteristics, disability severity is negatively correlated with both health utility and the MSQOL-54 PHCS, however not with MHCS. The correlation between disease severity and lower utility or physical health has been examined extensively [33–35], along with the negative effect of PPMS on HRQOL [36, 37]. Though one might expect that increasing disability will lead to lower mental health, this was not found, neither in the correlation of EQ-5D dimensions to EDSS or in the multivariate regression analysis. Symptoms such as depression, fatigue and anxiety are commonly known to have a negative effect on HRQOL [37, 38]. The psychological components of MS are just as important as the physical symptoms when managing HRQOL [39], though perhaps more difficult to target.

We want to inform physicians and policy makers that it is useful to include HRQOL instruments in clinical practice and in clinical trials. The use of such instruments in clinical practice enables physicians to know what dimensions of HRQOL to target. As such, it is possible to set up a personalized treatment plan, together with the patient, based on the HRQOL results [38]. Moreover, the use of HRQOL instruments in clinical care has shown significant benefits to the care given to patients [38]. Furthermore, we want to address the importance of measuring health utility and disease-specific HRQOL as end-points in clinical trials. Instruments such as the EQ-5D-5L and MSQOL-54 are able to quantity the potential added value of the new treatment or technology under examination, from the patient’s perspective, in terms of HRQOL [40]. This information is needed to perform a health technology assessment and the subsequent economic evaluation, which in turn is essential for health policy makers to decide on reimbursement decisions and allowing the treatment or technology to enter the market [6, 41].

### Limitations

There are various limitations to our study worth noting The design of this study was to examine HRQOL in Europe, and not between-country differences. However, post-hoc analysis revealed some between-country differences in HRQOL. Possible explanations of between country differences in HRQOL may be explained differing healthcare systems, the recruitment method and small samples in the UK and Spain. The healthcare systems of the examined countries differ, thereby possibly affecting the quality of care received by patients. Patients were recruited via the information channels of national patient societies, social media and via an MS specialist in France. Therefore, we had no control over how and to whom the patient societies reached out too, or the reach of our social media channels within the MS community. It is possible that the patient societies differed in how actively they promoted the study, leading to selection bias. Furthermore, the small samples from the United Kingdom and Spain limits their representability of the HRQOL status of those two countries, thus caution is needed when making statements on their HRQOL. Since we had small samples, we did not perform regressions per country. If larger samples had been recruited this may have given insight into possible country differences. Future studies should be designed to specifically examine cross-country differences and controlling for more information than we have included in this study.

Many disease-specific HRQOL instruments have been developed since the introduction of the MSQOL-54 in 1995, such as the Functional Assessment of MS (FAMS), the Hamburg Quality of Life Questionnaire in MS (HAQUAMS), the MS Impact Scale-29 (MSIS-29) and the MS International Quality of Life questionnaire (MusiQoL) to name a few [6], each focussing on a variety of MS-related domains. Though perhaps more relevant when examining only disease-specific HRQOL we did not find them suitable for this study. A deliberate choice was made to use the hybrid MSQOL-54 given that it is an extension of the generic SF-36, enabling comparability to other diseases. However, they are useful when examining a specific MS-specific HRQOL domain, and future researchers should include them when deemed necessary.

One MS centre in France was involved in the data collection and this may have led to sampling bias. This reduces the comparability of the French patients to patients in the other countries, although it is comparable to a previous French multicentre study by Lebrun-Frenay et al. (2017). For example, both studies found that patients were in their early thirties when diagnosed, at least 50% of the patients had a university degree and the type of DMT used was similar [42]. The similarities in the patient demographics show that similar recruitment methods lead to comparable patient populations, thereby validating the results of the study. Our findings give more in depth knowledge about the combined generic and disease-specific HRQOL status of French MS patients since Lebrun-Frenay and colleagues only examined health utility using the EQ-5D-3L.

Three patients (other than patients living elsewhere) filled out the survey in a language that differed from the official language of their country of residence which may have impacted health utility results (since health utility is somewhat influenced by the choice of national value set) [19, 43]. All patients were analysed based on country of residence, not on their nationality or user language of the survey (which may have differed across participants). However, since only involved three patients were involved, this had no major impact on the results.

Since the EDSS was self-reported, it is possible that some patients incorrectly estimated their EDSS due to a lack of understanding of the scale, despite having the option to indicate that they did not know it. However, the negative association we observed between EDSS and HRQOL suggests that this issue had a limited effect on the results. Nevertheless, caution is needed when interpreting the results. At the time of data collection the self-reported disability status scale (SRDSS) as a proxy measure to estimate EDSS was not yet available, however such a measure may be useful for self-assessment of disability in an online questionnaire environment [44]. Another measure that we could have employed was the self-reported Patient Determined Disease Steps, although this would have limited comparisons with other studies [45].

## Conclusions

Our results indicate that, till now, the effects of MS on HRQOL may have been underestimated in real-world MS patients. The combined use of both generic and disease-specific HRQOL instruments as outcome measures in clinical trials and observational studies allow for a deeper understanding about specific health needs of MS patients. To enhance the comparability of cross-country data from RCTs, observational studies, or patient registries it is essential to use the same instruments consequently. This study has made a first attempt to do so across Europe, however a more collective effort has to be made by all persons involved in health care research.

## Supplementary Information

**Additional file 1.**

## Data Availability

The data that support the findings of this study are available on request from the corresponding author. The data are not publicly available due to privacy or ethical restrictions. The code is available on request from the corresponding author.

## Abbreviations

ANOVA: Analysis of variance
CIS: Clinically isolated syndrome
DMT: Disease modifying therapy
EDSS: Expanded disability status scale
EQ-5D-3L: EuroQOL 5 dimensions questionnaire with 3 levels
EQ-5D-5L: EuroQOL 5 dimensions questionnaire with 5 levels
FAMS: Functional Assessment of MS
HAQUAMS: Hamburg Quality of Life Questionnaire in MS
HRQOL: Health related quality of life
INF-β: Interferon beta
MCS: Mental composite score
MHCS: Mental health composite score
MS: Multiple sclerosis
MSIS-29: MS Impact Scale-29
MSQOL-54: Multiple Sclerosis Quality of Life (MSQOL)-54 instrument
MusiQoL: MS International Quality of Life questionnaire
PCS: Physical composite score
PHCS: Physical health composite score
PPMS: Primary progressive multiple sclerosis
QOL: Quality of life
RCT: Randomized controlled trial
RRMS: Relapsing-remitting multiple sclerosis
SD: Standard deviation
SF-36: Medical Outcomes Study Short Form-36
SPMS: Secondary progressive multiple sclerosis
