# Supplementary material for: Health-related quality of life of multiple sclerosis patients: a European multi-country study
Source: Arch Public Health. 2021 Mar 20;79:39. doi: 10.1186/s13690-021-00561-z (PMC7980344; doi:10.1186/s13690-021-00561-z)
Supplement: Supplementary file 1 — Additional file 1. [file 13690_2021_561_MOESM1_ESM.docx]

**Appendix**

|  | **Total**  **(n=182)** | **The Netherlands (n=88)** | **France**  **(n=58)** | **The United Kingdom (n=15)** | **Spain**  **(n=10)** | **Elsewhere (n=11)** | **P-value^a^** |
| --- | --- | --- | --- | --- | --- | --- | --- |
| **INJECTABLES** |  |  |  |  |  |  |  |
| **INF-β 1a (Avonex), n (%)** |  |  |  |  |  |  | 0.018 |
| I am currently using this treatment | 7 (3.85) |  | 4 (6.90) | 1 (6.67) |  | 2 (18.18) |  |
| I have used this treatment in the past | 38 (20.88) | 16 (18.18) | 16 (27.59) | 1 (6.67) | 2 (20.00) | 3 (27.17) |  |
| I have never used this treatment | 137 (75.27) | 72 (81.82) | 38 (65.52) | 13 (86.67) | 8 (80.00) | 6 (54.55) |  |
| **INF-β 1a (Rebif) , n (%)** |  |  |  |  |  |  | 0.292 |
| I am currently using this treatment | 6 (3.30) | 1 (1.14) | 3 (5.17) | 1 (6.67) | 1 (10.00) |  |  |
| I have used this treatment in the past | 40 (21.98) | 19 (21.59) | 12 (20.69) | 2 (13.33) | 2 (20.00) | 5 (45.55) |  |
| I have never used this treatment | 136 (74.73) | 68 (77.27) | 43 (74.14) | 12 (80.00) | 7 (70.00) | 6 (54.55) |  |
| **PegINF-β 1a (Plegridy), n (%)** |  |  |  |  |  |  | 0.163 |
| I am currently using this treatment | 5 (2.75) | 2 (2.27) | 1 (1.72) |  | 2 (20.00) |  |  |
| I have used this treatment in the past | 11 (6.04) | 8 (9.09) | 2 (3.45) |  | 1 (10.00) |  |  |
| I have never used this treatment | 166 (91.21) | 78 (88.64) | 55 (94.83) | 15 (100.00) | 7 (70.00) | 11 (100.00) |  |
| **INF-β 1b (Extavia), n (%)** |  |  |  |  |  |  | 0.775 |
| I have used this treatment in the past | 3 (1.65) | 1(1.14) | 2 (3.45) |  |  |  |  |
| I have never used this treatment | 179 (98.35) | 87 (98.86) | 56 (96.55) | 15 (100.00) | 10 (100.00) | 11 (100.00) |  |
| **GA (Copaxone), n (%)** |  |  |  |  |  |  | 0.290 |
| I am currently using this treatment | 9 (4.95) | 4 (4.55) | 4 (6.90) |  |  | 1 (9.09) |  |
| I have used this treatment in the past | 54 (29.67) | 28 (31.82) | 21 (36.21) | 1 (6.67) | 2 (20.00) | 2 (18.18) |  |
| I have never used this treatment | 119 (65.38) | 56 (63.64) | 33 (56.90) | 14 (93.33) | 8 (80.00) | 8 (72.73) |  |
| **ORAL** |  |  |  |  |  |  |  |
| **DMF (Tecfidera), n (%)** |  |  |  |  |  |  | 0.877 |
| I am currently using this treatment | 18 (9.89) | 8 (9.09) | 6 (10.34) | 3 (20.00) |  | 1 (9.09) |  |
| I have used this treatment in the past | 23 (12.64) | 13 (14.77) | 6 (10.34) | 1 (6.67) | 2 (20.00) | 1 (9.09) |  |
| I have never used this treatment | 141 (77.47) | 67 (76.14) | 46 (79.31) | 11 (73.33) | 8 (80.00) | 9 (81.82) |  |
| **Teriflunomide (Aubagio), n (%)** |  |  |  |  |  |  | 0.240 |
| I am currently using this treatment | 14 (7.69) | 3 (3.41) | 8 (13.79) | 1 (6.67) | 1 (10.00) | 1 (9.09) |  |
| I have used this treatment in the past | 20 (10.99) | 12 (13.64) | 5 (8.62) |  | 1 (10.00) | 2 (18.18) |  |
| I have never used this treatment | 148 (81.32) | 73 (82.95) | 45 (77.59) | 14 (93.33) | 8 (80.00) | 8 (72.73) |  |
| **Cladribine (Mavenclad), n (%)** |  |  |  |  |  |  | 0.115 |
| I am currently using this treatment | 1 (0.55) |  |  |  |  | 1 (9.09) |  |
| I have never used this treatment | 181 (99.45) | 88 (100.00) | 58 (100.00) | 15 (100.00) | 10 (100.00) | 10 (90.91) |  |
| **Fingolimod (Gilenya), n (%)** |  |  |  |  |  |  | 0.554 |
| I am currently using this treatment | 19 (10.44) | 9 (10.23) | 7 (12.07) | 1 (6.67) |  | 2 (18.18) |  |
| I have used this treatment in the past | 13 (7.14) | 5 (5.68) | 5 (8.62) |  | 1 (10.00) | 2 (18.18) |  |
| I have never used this treatment | 150 (82.42) | 74 (84.09) | 46 (79.31) | 14 (93.33) | 9 (90.00) | 7 (63.64) |  |
| **INFUSION** |  |  |  |  |  |  |  |
| **Alemtuzumab (Lemtrada), n (%)** |  |  |  |  |  |  | 0.737 |
| I am currently using this treatment | 6 (3.30) | 4 (4.55) | 1 (1.72) | 1 (6.67) |  |  |  |
| I have never used this treatment | 176 (96.70) | 84 (95.45) | 57 (98.28) | 14 (93.33) | 10 (100.00) | 11 (100.00) |  |
| **Natalizumab (Tysabri), n (%)** |  |  |  |  |  |  | 0.265 |
| I am currently using this treatment | 8 (4.40) | 2 (2.27) | 3 (5.17) | 1 (6.67) | 1 (10.00) | 1 (9.09) |  |
| I have used this treatment in the past | 23 (12.64) | 11 (12.50) | 10 (17.24) |  |  | 2 (18.18) |  |
| I have never used this treatment | 151 (82.97) | 75 (85.23) | 45 (77.59) | 14 (93.33) | 9 (90.00) | 8 (72.73) |  |
| **Ocrelizumab (Ocrevus), n (%)** |  |  |  |  |  |  | 0.003 |
| I am currently using this treatment | 39 (21.43) | 29 (32.95) | 5 (8.62) |  | 3 (30.00) | 2 (18.18) |  |
| I have used this treatment in the past | 3 (1.65) | 1 (1.14) | 2 (3.45) |  |  |  |  |
| I have never used this treatment | 140 (76.92) | 58 (65.91) | 51 (87.93) | 15 (100.00) | 7 (70.00) | 9 (81.82) |  |

**Table A1. Current and past disease modifying treatment of the total study population and according to country of residence (the Netherlands, France, the United Kingdom, Spain and elsewhere (Germany or Italy)), 2019.**

**^a^**: Fisher’s exact test. INF-β: Interferon beta, PegINF- β: peginterferon beta, GA: glatiramer acetate, DMF: dimethyl fumarate.

|  | **EDSS** | **P-value** |
| --- | --- | --- |
| **Mobility** | 0.83 | <0.001 |
| **Self-care** | 0.68 | <0.001 |
| **Usual activities** | 0.73 | <0.001 |
| **Pain / discomfort** | 0.46 | <0.001 |
| **Anxiety / depression** | 0.06 | 0.55 |

**Table A2. Spearman correlation coefficient of the total study population (the Netherlands, France, the United Kingdom, Spain and elsewhere (Germany or Italy)), 2019.** EDSS: Expanded Disability Status Scale.

|  | **Mobility** | | | | | **Self-care** | | | | | **Usual activities** | | | | |
| --- | --- | --- | --- | --- | --- | --- | --- | --- | --- | --- | --- | --- | --- | --- | --- |
|  | No | Slight | Moderate | Severe | Unable to | No | Slight | Moderate | Severe | Unable to | No | Slight | Moderate | Severe | Unable to |
| **Age** |  | 0.046** | 0.081*** | 0.148*** | 0.148** |  | 0.039** | 0.033 | 0.068 | 0.163** |  | 0.017 | 0.060*** | 0.072*** | -0.042 |
| **Age at diagnosis** |  | 0.017 | 0.050** | 0.139*** | -0.021 |  | 0.048** | 0.049** | 0.057 | 0.048 |  | 0.003 | 0.026 | 0.058** | -0.023 |
| **Time since diagnosis** |  | 0.054* | 0.060** | 0.013 | 0.184*** |  | -0.015 | -0.033 | 0.017 | 0.108* |  | 0.030 | 0.066* | 0.029 | -0.054 |
| **Gender (male 0, female 1)** |  | -0.230 | -1.066** | -0.823 | -1.922* |  | -0.065 | -1.319** | -1.520 | 13.422 |  | 1.245 | 2.184* | 1.353 | 12.527 |
| **Marital status** |  |  |  |  |  |  |  |  |  |  |  |  |  |  |  |
| Single |  |  |  |  |  |  |  |  |  |  |  |  |  |  |  |
| Partnered |  | -1.098* | -1.098* | -2.890** | -1.791 |  | -0.827 | -0.174 | -15.293 | 14.316 |  | -0.074 | 0.099 | -0.806 | -15.165 |
| Married |  | -0.278 | -0.532 | -0.096 | -1.993 |  | -0.134 | -0.511 | -1.792 | 14.267 |  | -0.470 | 0.289 | -0.677 | -15.161 |
| Divorced |  | -0.406 | 0.325 | 0.693 | -12.290 |  | 0.966 | -14.555 | 1.253 | -0.169 |  | -0.241 | 15.575 | 14.285 | 15.671 |
| Widowed |  | 14.066 | -0.580 | -0.483 | -1.365 |  | 16.434 | -0.331 | -1.025 | 14.054 |  | 15.699 | 0.261 | -0.506 | -1.317 |
| **Educational level** |  |  |  |  |  |  |  |  |  |  |  |  |  |  |  |
| Primary education |  |  |  |  |  |  |  |  |  |  |  |  |  |  |  |
| Secondary education |  | 16.301 | 17.674 | 17.839 | 18.721 |  | 15.171 | 15.813 | 17.016 | 0.735 |  | 18.911 | 19.161 | 18.301 | 20.498 |
| Vocational/technical education |  | 17.705 | 17.615 | 17.685 | 3.169 |  | 14.908 | 14.857 | 0.761 | 14.556 |  | 18.976 | 19.102 | 19.177 | 18.889 |
| University |  | 17.286 | 17.089 | 17.072 | 17.586 |  | 14.141 | 13.866 | 14.377 | 14.665 |  | 18.036 | 17.830 | 17.791 | 17.503 |
| Other |  | 2.967 | 18.549 | 17.839 | 2.967 |  | 15.681 | 16.323 | 1.169 | 1.169 |  | 5.072 | 18.314 | 18.995 | 5.072 |
| **Type of MS** |  |  |  |  |  |  |  |  |  |  |  |  |  |  |  |
| CIS / RRMS |  |  |  |  |  |  |  |  |  |  |  |  |  |  |  |
| PPMS |  | 0.045^I^ | 17.173^I^ | 19.491^I^ | 19.571^a^ |  | 2.349*** | 3.305*** | 4.691*** | 18.701 |  | 0.121^I^ | 16.365^I^ | 18.082^a^ | 18.593^a^ |
| SPMS |  | 13.445^I^ | 14.926^I^ | 15.335^I^ | 17.207^a^ |  | 1.587*** | 1.108 | 2.900** | 16.909 |  | 15.352^I^ | 15.311^I^ | 15.797^a^ | 17.406^a^ |
| **Country of residence** |  |  |  |  |  |  |  |  |  |  |  |  |  |  |  |
| United Kingdom |  |  |  |  |  |  |  |  |  |  |  |  |  |  |  |
| France |  | -1.658 | -2.064* | -2.708** | 14.615 |  | -1.259* | -2.685*** | -17.599 | 15.501 |  | -0.318 | -1.181 | -2.110 | -16.481 |
| The Netherlands |  | -0.588 | -1.133 | -1.484 | 14.951 |  | -0.645 | -1.427** | -1.204 | 16.067 |  | -0.051 | -0.718 | -0.510 | -1.608 |
| Spain |  | -2.890* | -2.484* | -19.033 | -2.439 |  | -1.974 | -17.899 | -17.899 | -0.511 |  | -0.405 | -2.484* | -2.197 | -17.118 |
| Elsewhere |  | -1.099 | -2.079 | -18.230 | -1.636 |  | -1.281 | -17.755 | -17.755 | -0.366 |  | -0.693 | -1.791 | -2.197 | -16.899 |
| **EDSS levels** |  |  |  |  |  |  |  |  |  |  |  |  |  |  |  |
| EDSS <= 2.5 |  |  |  |  |  |  |  |  |  |  |  |  |  |  |  |
| EDSS 3 – 5.5 |  | 2.351*** | 4.025*** | 18.380 | 19.262 |  | 2.447*** | 1.841 | 16.057 | 0.620 |  | 16.477 | 17.681 | 18.086 | 33.342 |
| EDSS >= 6 |  | 0.803 | 19.739 | 36.818 | 37.124 |  | 4.344*** | 4.586*** | 19.053 | 18.935 |  | 15.910 | 18.523 | 21.183 | 35.666 |
| Unknown/missing |  | 1.192** | 2.108*** | 17.443 | 1.482 |  | 1.732** | 1.984* | 0.472 | 15.389 |  | 0.824 | 1.801*** | 2.890** | 1.526 |
| **Current line of treatment** |  |  |  |  |  |  |  |  |  |  |  |  |  |  |  |
| Treatment naive |  |  |  |  |  |  |  |  |  |  |  |  |  |  |  |
| 1^st^-line DMT |  | 1.157 | 0.658 | -1.952 8* | -14.926 ^a^ |  | -1.567 | -1.750 | -17.561^a^ | -0.054 ^a^ |  | 1.609 | -0.134 | -1.569* | -14.494^a^ |
| 2^nd^-line DMT |  | 2.088* | 1.647 | -0.638 | -0.956 |  | -0.792 | -0.811 | -17.281^a^ | 14.681^a^ |  | 1.852 | 0.811 | -0.471 | -0.287^a^ |
| Treatment experience but currently no DMT |  | 1.860 | 1.368 | -0.807 | 0.356 |  | -0.734 | -1.204 | -2.303* | 16.185 ^a^ |  | 1.280 | 0.531 | -0.512 | -14.115^a^ |
| **** p<0.01, ** p<0.05, * p<0.1* | | | | | | | | | | | | | | | |

**Table A3 Predictors of EQ-5D-5L dimensions (univariate regression analysis) of the total study population (the Netherlands, France, the United Kingdom, Spain and elsewhere (Germany or Italy)), 2019.** CIS: clinically isolated syndrome; RRMS: relapsing-remitting multiple sclerosis; PPMS: primary progressive multiple sclerosis; SPMS: secondary progressive multiple sclerosis; EDSS: Expanded Disability Status Scale; DMT: disease modifying therapy. **^a^**: due to small sample size the model was not able to converge (very large standard errors).

|  | **Pain / discomfort** | | | | | **Anxiety / depression** | | | | | **Health utility** Univariate ^a^ | **Health utility**  Multivariate ^a^ | **Health utility** Univariate ^b^ | **Health utility**  Multivariate ^b^ |
| --- | --- | --- | --- | --- | --- | --- | --- | --- | --- | --- | --- | --- | --- | --- |
|  | No | Slight | Moderate | Severe | Unable to | No | Slight | Moderate | Severe | Unable to |  |  |  |  |
| **Age** |  | 0.033 | 0.076*** | 0.069** | 0.021 |  | -0.005 | -0.005 | -0.026 | 0.034 | -0.006*** |  | -0.005*** |  |
| **Age at diagnosis** |  | 0.022 | 0.059** | 0.042 | 0.060 |  | -0.009 | 0.007 | -0.006 | 0.052 | -0.007** |  | -0.005*** |  |
| **Time since diagnosis** |  | 0.024 | 0.034 | 0.049 | -0.194 |  | 0.005 | -0.023 | -0.043 | -0.039 | -0.000 |  | -0.001 |  |
| **Gender (male 0, female 1)** |  | -0.111 | 0.357 | -0.154 | -0.154 |  | -0.035 | 1.030* | 0.385 | 13.510 | 0.055 |  | 0.020 |  |
| **Marital status** |  |  |  |  |  |  |  |  |  |  |  |  |  |  |
| Single |  |  |  |  |  |  |  |  |  |  |  |  | 0.158*** |  |
| Partnered |  | -0.762 | -0.734 | -1.609 | -0.916 |  | 0.270 | 0.328 | -0.876 | -15.261 | 0.119* |  | 0.106** | 0.109** |
| Married |  | -0.028 | 0.182 | -0.613 | -14.691 |  | 0.613 | 0.328 | -0.588 | -1.568 | 0.084 |  | -0.037 | 0.103** |
| Divorced |  | -1.098 | -0.916 | 0.695 | -16.064 |  | -0.847 | -0.693 | -15.349 | -15.984 | -0.070 |  | 0.252 |  |
| Widowed |  | -0.267 | 15.699 | -0.622 | -1.216 |  | -15.295 | -15.441 | -16.219 | -16.855 | 0.171 |  |  |  |
| **Educational level** |  |  |  |  |  |  |  |  |  |  |  |  |  |  |
| Primary education |  |  |  |  |  |  |  |  |  |  |  |  |  |  |
| Secondary education |  | 0.267 | -14.397 | 1.315 | -12.494 |  | -16.088 | 0.013 | -0.307 | -12.888 | -0.296 |  | -0.289 |  |
| Vocational/technical education |  | 0.002 | -15.601 | 0.399 | 0.706 |  | -16.183 | -0.392 | -0.066 | -0.277 | -0.223 |  | -0.236 |  |
| University |  | -0.078 | -15.224 | -0.647 | -0.863 |  | -15.583 | 0.196 | 0.008 | 0.356 | -0.119 |  | -0.165 |  |
| Other |  | 13.410 | -1.071 | 0.000 | 0.000 |  | -14.701 | 1.400 | 1.484 | -12.050 | -0.160 |  | -0.258 |  |
| **Type of MS** |  |  |  |  |  |  |  |  |  |  |  |  |  |  |
| CIS / RRMS |  |  |  |  |  |  |  |  |  |  |  |  |  |  |
| PPMS |  | 0.634 | 1.160 | 1.561 | 3.219** |  | -0.653 | 0.098 | 1.492** | 1.674 | -0.387*** | -0.275*** | -0.394*** | -0.226*** |
| SPMS |  | 0.857 | 1.314 | 1.560 | -10.382 |  | 0.782 | 0.792 | -12.491^c^ | -12.434^c^ | -0.143** |  | -0.136** |  |
| **Country of residence** |  |  |  |  |  |  |  |  |  |  |  |  |  |  |
| United Kingdom |  |  |  |  |  |  |  |  |  |  |  |  |  |  |
| France |  | 0.140 | -0.356 | -0.762 | -0.916 |  | 0.811 | 0.251 | 1.232 | 15.675 | 0.281*** | 0.126*** | 0.096 |  |
| The Netherlands |  | -0.923 | -1.406 | -1.041 | -2.833 |  | -0.521 | -0.827 | -0.134 | -0.330 | 0.098 |  | 0.092 |  |
| Spain |  | -1.386 | -1.504 | -15.979 | -16.861 |  | 0.118 | -0.288 | 0.405 | 0.013 | 0.293*** |  | 0.250** |  |
| Elsewhere |  | 0.223 | -0.405 | -1.098 | -15.480 |  | 0.405 | 0.917 | -13.625 | 0.064 | 0.195* |  | 0.189* |  |
| **EDSS levels** |  |  |  |  |  |  |  |  |  |  |  |  |  |  |
| EDSS <= 2.5 |  |  |  |  |  |  |  |  |  |  |  |  |  |  |
| EDSS 3 -5.5 |  | 1.099 | 2.190** | 2.110** | 14.912 |  | -0.351 | -0.100 | -1.044 | -13.796 | -0.161*** | -0.129*** | -0.157*** | -0.126** |
| EDSS >= 6 |  | 0.916 | 2.516** | 3.314*** | 14.911 |  | 0.236 | 0.528 | 1.376 | -13.346 | -0.485*** | -0.383*** | -0.458*** | -0.376*** |
| Unknown / missing |  | -0.123 | 1.155** | 0.644 | 12.346 |  | -0.332 | 0.542 | 0.524 | 0.236 | -0.151*** | -0.113*** | -0.138*** | -0.130*** |
| **Current line of treatment** |  |  |  |  |  |  |  |  |  |  |  |  |  |  |
| Treatment naive |  |  |  |  |  |  |  |  |  |  |  |  |  |  |
| 1^st^-line DMT |  | -0.223 | -0.270 | -1.992* | -1.705 |  | 1.600* | 1.482* | 0.788 | -13.305^c^ | 0.259 *** |  | 0.204*** |  |
| 2^nd^-line DMT |  | 0.145 | -0.022 | -0.251 | -0.811 |  | 1.300 | 0.943 | 1.076 | -1.004 | 0.115 |  | 0.100 |  |
| Treatment experience but currently no DMT |  | -0.328 | -0.041 | -0.223 | -14.253^c^ |  | 1.369 | 0.675 | 1.145 | -0.242 | 0.127 * |  | 0.080 |  |

**Table A3 (continued). Predictors of EQ-5D-5L dimensions (univariate regression analysis) of the total study population (the Netherlands, France, the United Kingdom, Spain and elsewhere (Germany or Italy)), 2019.** CIS: clinically isolated syndrome; RRMS: relapsing-remitting multiple sclerosis; PPMS: primary progressive multiple sclerosis; SPMS: secondary progressive multiple sclerosis; EDSS: Expanded Disability Status Scale; DMT: disease modifying therapy. ^a^ Regression with the utility calculated using the country-specific tariffs; ^b^ Regression was re-run with the utility calculated using only the Dutch tariffs; ^c^: due to small sample size the model was not able to converge (very large standard errors).

|  | **PH** | **HP** | **E** | **RLPP** | **P** | **SFMF** | **HD** | **PHCS**  Univariate | **PHCS**  Multivariate |
| --- | --- | --- | --- | --- | --- | --- | --- | --- | --- |
| **Age** | -1.192*** | -0.005 | 0.116 | -0.156 | -0.351* | -0.305 | 0.086 | -0.257** |  |
|  | (0.198) | (0.160) | (0.140) | (0.264) | (0.180) | (0.191) | (0.174) | (0.120) |  |
| **Age at diagnosis** | -0.905*** | 0.105 | 0.104 | -0.017 | -0.230 | -0.067 | -0.084 | -0.159 |  |
|  | (0.210) | (0.162) | (0.143) | (0.269) | (0.184) | (0.196) | (0.177) | (0.124) |  |
| **Time since diagnosis** | -0.577** | -0.194 | 0.028 | -0.254 | -0.234 | -0.437* | 0.304 | -0.190 |  |
|  | (0.289) | (0.215) | (0.190) | (0.356) | (0.245) | (0.257) | (0.234) | (0.163) |  |
| **Gender (male 0, female 1)** | 9.487* | -5.885 | -1.777 | 5.119 | -4.604 | -0.794 | -0.395 | 0.482 |  |
|  | (5.482) | (4.062) | (3.601) | (6.767) | (4.635) | (4.934) | (4.428) | (3.098) |  |
| **Marital status** |  |  |  |  |  |  |  |  |  |
| Single |  |  |  |  |  |  |  |  |  |
| Partnered | 16.484** | -0.724 | -2.249 | -3.671 | 6.435 | 6.030 | 7.579 | 3.770 |  |
|  | (6.818) | (5.111) | (4.512) | (8.439) | (5.811) | (6.235) | (5.558) | (3.923) |  |
| Married | 3.771 | 6.901 | 2.527 | 9.549 | 8.067 | 3.008 | 10.937** | 5.372 |  |
|  | (5.866) | (4.398) | (3.882) | (7.262) | (5.000) | (5.379) | (4.782) | (3.384) |  |
| Divorced | -16.942 | 4.435 | -8.439 | -9.226 | -10.747 | 1.672 | 13.056 | -4.081 |  |
|  | (12.399) | (9.295) | (8.205) | (15.349) | (10.568) | (11.279) | (10.108) | (7.097) |  |
| Widowed | -14.799 | -10.208 | 16.704 | -27.083 | -5.509 | 37.379 | -1.944 | -3.549 |  |
|  | (30.431) | (22.812) | (20.137) | (37.670) | (25.937) | (27.629) | (24.808) | (17.383) |  |
| **Educational level** |  |  |  |  |  |  |  |  |  |
| Primary education |  |  |  |  |  |  |  |  |  |
| Secondary education | -69.872** | -27.308 | -34.154* | -78.846** | -16.667 | -42.960 | -30.000 | -38.651** | -9.409** |
|  | (30.548) | (23.450) | (20.378) | (37.970) | (26.694) | (27.865) | (25.689) | (17.450) | (4.490) |
| Vocational/technical education | -50.265* | -26.051 | -39.806** | -78.109** | -14.005 | -21.896 | -30.597 | -33.782** | -6.424** |
|  | (29.656) | (22.765) | (19.783) | (36.861) | (25.914) | (27.051) | (24.938) | (16.940) | (2.471) |
| University | -38.257 | -21.478 | -33.719* | -63.070* | -7.579 | -26.601 | -30.088 | -27.890 |  |
|  | (29.592) | (22.716) | (19.740) | (36.781) | (25.858) | (26.994) | (24.884) | (16.905) |  |
| Other | -50.833 | -28.889 | -43.333** | -58.333 | -16.944 | -23.613 | -28.333 | -32.622* |  |
|  | (31.796) | (24.408) | (21.210) | (39.521) | (27.784) | (29.003) | (26.738) | (18.163) |  |
| **Type of MS** |  |  |  |  |  |  |  |  |  |
| CIS / RRMS |  |  |  |  |  |  |  |  |  |
| PPMS | -44.048*** | -13.521** | -9.258* | -25.467*** | -18.341*** | -21.186*** | -27.839*** | -21.055*** | -14.799*** |
|  | (6.858) | (5.712) | (5.062) | (9.401) | (6.460) | (6.983) | (5.975) | (4.219) | (4.043) |
| SPMS | -32.284*** | -5.580 | 2.036 | -12.232 | -7.753 | -8.781 | 0.102 | -9.204** |  |
|  | (6.858) | (5.712) | (5.062) | (9.401) | (6.460) | (6.795) | (5.975) | (4.105) |  |
| **Country of residence** |  |  |  |  |  |  |  |  |  |
| United Kingdom |  |  |  |  |  |  |  |  |  |
| France | 35.110*** | 1.406 | 2.941 | 18.391* | 9.272 | 11.086 | -2.655 | 10.251** |  |
|  | (8.347) | (6.455) | (5.607) | (10.607) | (7.410) | (7.901) | (7.080) | (4.968) |  |
| The Netherlands | 20.805** | 3.891 | 11.488** | 9.375 | 14.331** | 20.771*** | 3.326 | 10.174** | 5.364** |
|  | (8.049) | (6.225) | (5.408) | (10.228) | (7.146) | (7.635) | (6.828) | (4.800) | (2.445) |
| Spain | 43.259*** | 19.639** | 16.733** | 43.333*** | 18.111* | 27.266** | 16.500* | 23.758*** | 11.074*** |
|  | (11.763) | (9.098) | (7.903) | (14.948) | (10.444) | (10.986) | (9.979) | (6.907) | (5.002) |
| Elsewhere | 41.259*** | 14.222 | 16.533** | 21.970 | 17.172* | 21.819** | 4.727 | 18.075*** |  |
|  | (11.438) | (8.846) | (7.684) | (14.535) | (10.155) | (10.691) | (9.703) | (6.722) |  |
| **EDSS levels** |  |  |  |  |  |  |  |  |  |
| EDSS <= 2.5 |  |  |  |  |  |  |  |  |  |
| EDSS 3 – 5.5 | -26.887*** | -11.319** | -8.875** | -22.460*** | -9.201 | -8.020 | -4.794 | -12.436*** | -11.320*** |
|  | (5.574) | (4.833) | (4.396) | (8.171) | (5.635) | (5.929) | (5.465) | (3.482) | (3.419) |
| EDSS >= 6 | -60.314*** | -22.660*** | -13.150*** | -29.915*** | -20.540*** | -23.782*** | -16.735*** | -25.362*** | -21.194*** |
|  | (6.092) | (5.282) | (4.805) | (8.931) | (6.159) | (6.562) | (5.973) | (3.854) | (3.997) |
| Unknown / missing | -20.418*** | -15.286*** | -9.198** | -18.823*** | -2.682 | -12.847** | -7.077 | -11.534*** | -10.677*** |
|  | (4.652) | (4.033) | (3.669) | (6.819) | (4.703) | (4.949) | (4.561) | (2.906) | (2.851) |
| **Current line of treatment** |  |  |  |  |  |  |  |  |  |
| Treatment naive |  |  |  |  |  |  |  |  |  |
| 1^st^-line DMT | 29.700*** | 2.793 | 2.744 | 20.250** | 12.574* | 6.233 | 8.611 | 10.778** |  |
|  | (8.072) | (6.221) | (5.517) | (10.234) | (7.053) | (7.687) | (6.806) | (4.785) |  |
| 2^nd^-line DMT | 11.634 | -3.869 | 1.165 | 8.508 | 7.048 | 9.643 | 7.127 | 4.477 |  |
|  | (7.882) | (6.074) | (5.387) | (9.993) | (6.886) | (7.514) | (6.646) | (4.677) |  |
| Treatment experienced but currently no DMT | 11.422 | 1.164 | 2.235 | 9.559 | 2.892 | 6.098 | 7.549 | 4.857 |  |
|  | (8.694) | (6.700) | (5.942) | (11.022) | (7.596) | (8.252) | (7.331) | (5.137) |  |
| **** p<0.01, ** p<0.05, * p<0.1 Standard errors are in parenthesis* | | | | | | | | | |

**Table A4. Predictors of MSQOL-54 scales (univariate regression analysis) of the total study population (the Netherlands, France, the United Kingdom, Spain and elsewhere (Germany or Italy)), 2019..** PF: physical function, HP: health perception, E: energy / fatigue, RLP: role limitation physical, P: pain, SFMF: sexual function male and female combined, HD: health distress, PHCS: physical health composite score; CIS: clinically isolated syndrome; RRMS: relapsing-remitting multiple sclerosis; PPMS: primary progressive multiple sclerosis; SPMS: secondary progressive multiple sclerosis; EDSS: Expanded Disability Status Scale; DMT: disease modifying therapy.

|  | **HD** | **OQOL** | **EWB** | **RLEP** | **CF** | **MHCS**  Univariate | **MHCS**  Multivariate |
| --- | --- | --- | --- | --- | --- | --- | --- |
| **Age** | 0.086 | -0.152 | 0.292** | 0.148 | 0.356** | 0.149 |  |
|  | (0.174) | (0.134) | (0.147) | (0.312) | (0.174) | (0.154) |  |
| **Age at diagnosis** | -0.084 | -0.295** | 0.166 | -0.072 | 0.245 | -0.010 |  |
|  | (0.177) | (0.135) | (0.151) | (0.316) | (0.178) | (0.156) |  |
| **Time since diagnosis** | 0.304 | 0.244 | 0.240 | 0.390 | 0.217 | 0.282 |  |
|  | (0.234) | (0.181) | (0.200) | (0.416) | (0.237) | (0.205) |  |
| **Gender (male 0, female 1)** | -0.395 | 2.534 | -7.209* | -8.626 | -0.139 | -3.742 |  |
|  | (4.428) | (3.457) | (3.768) | (7.857) | (4.517) | (3.883) |  |
| **Marital status** |  |  |  |  |  |  |  |
| Single |  |  |  |  |  |  |  |
| Partnered | 7.579 | 7.036 | 4.337 | 11.485 | 2.791 | 6.816 |  |
|  | (5.558) | (4.334) | (4.784) | (10.071) | (5.663) | (4.958) |  |
| Married | 10.937** | 6.063 | 5.726 | 10.989 | 6.082 | 7.889* |  |
|  | (4.782) | (3.730) | (4.116) | (8.712) | (4.873) | (4.289) |  |
| Divorced | 13.056 | 5.911 | 11.885 | 4.342 | 13.386 | 9.444 |  |
|  | (10.108) | (7.883) | (8.701) | (18.118) | (10.299) | (8.921) |  |
| Widowed | -1.944 | 6.618 | 14.028 | 51.961 | -5.185 | 16.736 |  |
|  | (24.808) | (19.347) | (21.353) | (44.290) | (25.277) | (21.807) |  |
| **Educational level** |  |  |  |  |  |  |  |
| Primary education |  |  |  |  |  |  |  |
| Secondary education | -30.000 | -12.192 | -23.077 | -38.462 | -5.000 | -23.068 |  |
|  | (25.689) | (19.889) | (21.752) | (45.404) | (25.866) | (22.460) |  |
| Vocational/technical education | -30.597 | -11.283 | -22.627 | -45.274 | -9.080 | -25.104 |  |
|  | (24.938) | (19.308) | (21.116) | (44.078) | (25.111) | (21.803) |  |
| University | -30.088 | -8.393 | -27.537 | -43.369 | -2.719 | -24.458 |  |
|  | (24.884) | (19.266) | (21.070) | (43.987) | (25.056) | (21.759) |  |
| Other | -28.333 | -14.467 | -34.000 | -27.778 | -10.833 | -24.722 |  |
|  | (26.738) | (20.701) | (22.640) | (47.258) | (26.923) | (23.377) |  |
| **Type of MS** |  |  |  |  |  |  |  |
| CIS / RRMS |  |  |  |  |  |  |  |
| PPMS | -27.839*** | -20.153*** | -13.220** | -22.012** | -2.471 | -17.066*** | -17.815*** |
|  | (5.975) | (4.665) | (5.300) | (10.971) | (6.361) | (5.343) | (5.326) |
| SPMS | 0.102 | -0.003 | 4.545 | 19.165* | 8.706 | 7.183 |  |
|  | (5.975) | (4.665) | (5.300) | (10.971) | (6.361) | (5.343) |  |
| **Country of residence** |  |  |  |  |  |  |  |
| United Kingdom |  |  |  |  |  |  |  |
| France | -2.655 | 7.724 | -6.638 | -5.380 | -6.822 | -3.051 |  |
|  | (7.080) | (5.451) | (5.837) | (12.566) | (7.233) | (6.137) |  |
| The Netherlands | 3.326 | 12.868** | 8.091 | 9.885 | -1.807 | 7.168 |  |
|  | (6.828) | (5.257) | (5.629) | (12.106) | (6.975) | (5.912) |  |
| Spain | 16.500* | 15.975** | 6.000 | 16.667 | 0.500 | 11.000 |  |
|  | (9.979) | (7.683) | (8.227) | (17.678) | (10.193) | (8.633) |  |
| Elsewhere | 4.727 | 13.176* | 6.636 | -4.848 | -7.545 | 2.663 |  |
|  | (9.703) | (7.470) | (7.999) | (17.189) | (9.912) | (8.395) |  |
| **EDSS levels** |  |  |  |  |  |  |  |
| EDSS <= 2.5 |  |  |  |  |  |  |  |
| EDSS 3 – 5.5 | -4.794 | -5.191 | 3.327 | 3.961 | -8.878 | -1.415 |  |
|  | (5.465) | (4.149) | (4.730) | (9.875) | (5.560) | (4.847) |  |
| EDSS >= 6 | -16.735*** | -17.857*** | -4.044 | -0.874 | -3.041 | -7.789 |  |
|  | (5.973) | (4.535) | (5.170) | (10.785) | (6.077) | (5.294) |  |
| Unknown / missing | -7.077 | -5.545 | -3.781 | -8.293 | -10.202** | -7.121* |  |
|  | (4.561) | (3.463) | (3.947) | (8.279) | (4.640) | (4.064) |  |
| **Current line of treatment** |  |  |  |  |  |  |  |
| Treatment naive |  |  |  |  |  |  |  |
| 1^st^-line DMT | 8.611 | 8.965* | -3.940 | -0.068 | 0.328 | 1.709 |  |
|  | (6.806) | (5.226) | (5.831) | (12.062) | (6.845) | (5.970) |  |
| 2^nd^-line DMT | 7.127 | 4.424 | -3.853 | 6.683 | -7.185 | 1.259 |  |
|  | (6.646) | (5.103) | (5.693) | (11.809) | (6.684) | (5.844) |  |
| Treatment experienced but currently no DMT | 7.549 | 9.750* | -1.706 | 7.843 | -5.000 | 3.450 |  |
|  | (7.331) | (5.628) | (6.280) | (12.991) | (7.372) | (6.429) |  |
| **** p<0.01, ** p<0.05, * p<0.1 Standard errors are in parenthesis* | | | | | | | |

**Table A4 (continued). Predictors of MSQOL-54 scales (univariate regression analysis) of the total study population (the Netherlands, France, the United Kingdom, Spain and elsewhere (Germany or Italy)), 2019.** HD: health distress, OQOL: overall quality of life, EWB: emotional well-being, RLE: role limitation emotional, CF: cognitive function, MHCS: mental health composite score; CIS: clinically isolated syndrome; RRMS: relapsing-remitting multiple sclerosis; PPMS: primary progressive multiple sclerosis; SPMS: secondary progressive multiple sclerosis; EDSS: Expanded Disability Status Scale; DMT: disease modifying therapy.
